# From fair predictions to just decisions? Conceptualizing algorithmic fairness and distributive justice in the context of data-driven decision-making

**DOI:** 10.3389/fsoc.2022.883999

**Published:** 2022-10-10

**Authors:** Matthias Kuppler, Christoph Kern, Ruben L. Bach, Frauke Kreuter

**Affiliations:** ^1^Department of Social Sciences, University of Siegen, Siegen, Germany; ^2^School of Social Sciences, University of Mannheim, Mannheim, Germany; ^3^Joint Program in Survey Methodology, University of Maryland, College Park, MD, United States; ^4^Department of Statistics, LMU Munich, Munich, Germany

**Keywords:** automation, prediction, algorithm, fairness, distributive justice

## Abstract

Prediction algorithms are regularly used to support and automate high-stakes policy decisions about the allocation of scarce public resources. However, data-driven decision-making raises problems of algorithmic fairness and justice. So far, fairness and justice are frequently conflated, with the consequence that distributive justice concerns are not addressed explicitly. In this paper, we approach this issue by distinguishing (a) fairness as a property of the algorithm used for the prediction task from (b) justice as a property of the allocation principle used for the decision task in data-driven decision-making. The distinction highlights the different logic underlying concerns about fairness and justice and permits a more systematic investigation of the interrelations between the two concepts. We propose a new notion of algorithmic fairness called error fairness which requires prediction errors to not differ systematically across individuals. Drawing on sociological and philosophical discourse on local justice, we present a principled way to include distributive justice concerns into data-driven decision-making. We propose that allocation principles are just if they adhere to well-justified distributive justice principles. Moving beyond the one-sided focus on algorithmic fairness, we thereby make a first step toward the explicit implementation of distributive justice into data-driven decision-making.

## 1. Introduction

In 2019, the United States signed into law the Foundations of Evidence-based Policy Act (Hart and Yohannes, 2019) which requires government agencies to exploit available evidence and data when making policy decisions. Similar initiatives are under way in other countries. The German government, for example, pledged over two hundred million euro to build data labs in every ministry to improve decision-making and bring data-driven evidence into everyday policy-making (Engler, 2022b). Prediction algorithms play an increasingly important role in meeting evidence-based policy-making goals in settings where policies affect the allocation of social benefits and interventions to individuals. In these settings, algorithms are used to predict the likelihood of a risk in order to target an intervention or help.

Carton et al. (2016), for instance, predicted the risk of adverse behavior among police officers and used the predicted risk scores to prioritize preventive training and counseling. The New Zealand government used prediction algorithms and historic data about families to identify new-born children who are at high risk for maltreatment and, hence, are prioritized for preventive services (New Zealand Ministry of Social Development, 2014). More recently, prediction models were used to support COVID-19 prevention and treatment decisions in Israel (Barda et al., 2020).

The adoption of prediction algorithms reflects a long-standing trend toward less discretionary, data-driven decision procedures (Elster, 1992). Data-driven approaches promise to render decision-making processes more accurate and evidence-based and, by limiting decision-maker discretion, less susceptible to human biases and manipulation (Lepri et al., 2018). In domains with profound impacts on life chances, including decisions regarding policing (Alikhademi et al., 2021), welfare benefits (Desiere et al., 2019), and criminal justice (Angwin et al., 2016), concerns are raised that prediction algorithms, despite the gained efficiencies, can inherit human biases and perpetuate unfair discrimination against vulnerable and historically disadvantaged groups (Barocas and Selbst, 2016). Such perpetuation is particularly likely when prediction algorithms are based on data where (a) key groups are misrepresented or missing, (b) outcomes are systematically mislabeled, and (c) past discriminatory behavior is recorded and creates historical bias (Rodolfa et al., 2021). These concerns are fundamental to the discussion of an AI Act for the European Union (Engler, 2022a).

To address these challenges and to guide the design of non-discriminatory prediction algorithms, the research community developed formal fairness definitions—called *fairness metrics*—that quantify the extent to which model predictions satisfy various notions of fairness (Makhlouf et al., 2020; Mitchell et al., 2021). Independence, for instance, states that predictions are fair if they are statistically independent from a pre-defined set of protected attributes like sex or disability. Disagreement exists over which metric captures the underlying concern about fairness best. The debate is exacerbated by the fact that some fairness definitions are incompatible, such that a prediction model cannot satisfy all definitions simultaneously (Chouldechova, 2016; Kleinberg et al., 2016). Recent research attempted to resolve this conundrum by identifying the moral assumptions underlying the different fairness definitions and delineating the situations in which certain assumptions are (not) justified (Heidari et al., 2019; Friedler et al., 2021).

In this paper, we propose an alternative approach to fairness and justice in data-driven decision-making. Existing fairness approaches tend to mix technical concerns about the statistical properties of algorithmic predictions with moral concerns about the justice of decisions that are based on these predictions. To highlight the distinction between technical and moral concerns, we define fairness as a property of the prediction algorithm and justice as a property of the decision rule. From this perspective, there is little room for moral debate at the prediction step. Predictions should represent the true underlying values of the prediction target as accurately as possible for all candidates to which the prediction algorithm is applied. No candidate should have a disproportionate risk of an erroneous prediction that systematically depends on her characteristics. We call this notion *error fairness* and define it as the requirement that prediction errors are not systematically related to observed and unobserved features of the candidates. While this perspective is immanent in the (multi-group) fairness notions of Kim et al. (2019) and Hebert-Johnson et al. (2018), we highlight that the common group-based approach to algorithmic fairness is unable to guarantee error fairness. Suggestions for metrics that capture error fairness are made.

We define the decision step as a problem of local justice (Elster, 1992). Local justice focuses on the principles that organizations use to allocate benefits and burdens—a focus that aligns well with the scope of data-driven decision-making. The selection of allocation principles is informed by middle-range distributive justice principles. We consider four justice principles: equality, desert, need, and efficiency (Deutsch, 1975; Konow, 2003; Törnblom and Kazemi, 2015). Each justice principle defines a class of criteria that should guide the allocation of benefits and burdens.

We make two contributions to the literature on algorithmic fairness and justice. First, we clarify the relation between fairness and justice in data-driven decision-making and provide a clear definition of both concepts. Second, we provide an overview of distributive justice principles and a recipe for implementing the principles into the decision-making pipeline. Taken together, our approach guides the design of data-driven decision procedures that go from fair predictions to just decisions.

The argument proceeds as follows: Section 2 defines the class of decision problems that we deal with in this paper and introduces our definitions of justice and fairness. Section 3 elaborates on the problem of justice in data-driven decision-making from within the framework of local justice. Section 4 discusses the problem of fairness in data-driven decision-making and introduces the notion of error fairness. Section 5 provides a broader picture of the problem of bias in data-driven decision-making that is inspired by the distinction between justice and fairness. Section 6 concludes with a discussion of the practical implications and limitations of our approach.

## 2. Problem statement: Data-driven decisions, justice, and fairness

### 2.1. Decision problem

Our entire argument deals with the following decision problem: Consider an institution with a fixed amount *X* ∈ ℕ_+_ of a good that it can allocate among a fixed number of *i* = 1, …, *n* candidates. The institution must decide which candidates should receive a unit the good. Goods are material and immaterial things that can be attached to or owned by the candidates. Goods can be valued positively (as something one would like to have) or negatively (as something one would like to avoid). Positively valued goods are benefits, negatively valued goods are burdens. Because of the symmetry between benefits and burdens (exemption from a burden is a benefit and vice versa), we use the general term *good* in the following^1^.

Candidates are the actors who are eligible for the good. Candidates can be individual (e.g., humans or animals) and corporate (e.g., organizations or sub-units of organizations) actors. The pool of eligible candidates is usually specific to the allocating institution. For instance, not every citizen is eligible for participation in the labor market activation programs allocated by a public employment agency. Similarly, only sub-units of a firm are eligible for the allocation of resources by the central governance unit of the firm.

The decision problem is further characterized by scarcity, indivisibility, homogeneity, and rivalry. *Scarcity* means that the number of candidates (demand for the good) exceeds the number of units of the good that can be allocated (supply of the good). Scarcity may be natural (there is no way to increase supply) or artificial (supply could be increased at the cost of decreasing supply of another good). Paintings by Pablo Picasso are a naturally scarce good. Prison sentencing is an artificially scarce good. Courts could exempt every defendant from the burden of a prison sentence at the cost of reducing the overall safety of society. *Indivisibility* means that the good comes in fixed units that cannot be sub-divided any further—at least not without losing value or getting destroyed. Kidney transplants, for instance, are indivisible. One cannot (at least currently) transplant one kidney into two patients. *Homogeneity* means that only one version of the good exists and that any two units of the good are indistinguishable. *Rivalry* means that ownership of the good by one candidate A precludes ownership of the good by any other candidate B, C, … now and in the future unless the good is re-allocated.

Finally, we focus on *binary decisions*. For each candidate, the institution decides between two options: allocate one unit of the good to the candidate (positive decision) or allocate no unit of the good to the candidate (negative decision). The decision problem amounts to selecting the subset n^*^ ⊂ *n* of candidates who receive the good. In this paper, we do not consider decisions about the number of units of the good allocated to each candidate. In principle, however, our approach could be extended to such decisions.

The task of the institution is to formulate an *allocation principle*. An allocation principle is a rule that defines how goods are allocated to candidates. The principle defines a set of decision-relevant criteria and specifies the relationship between the criteria and the allocation of goods. In most cases, the decision-relevant criteria are attributes of the candidates. Allocation principles differ in the amount of discretion awarded to human decision-makers, varying from informal open-ended (high discretion) to formal rule-based (low discretion) principles.

In this paper, we focus on formal rule-based allocation principles because the impetus for implementing data-driven decision-making is usually a desire to reduce human discretion. For the most part, we rely on *ranking-based allocation principles*. Candidates are brought into a rank order based on the value they have on the decision-relevant criterion. At the top of the rank order are the candidates who, according to their value on the decision criterion, have the strongest claim to the good. If there are *X* units of the good, each of the *X* top-ranked candidates receives one unit of the good^2^. A bank, for instance, might allocate loans (the good) based on candidates' history of loan repayment (decision criterion). If the bank can allocate *X* = 10 loans among *n* = 100 applicants, it will allocate the loan to the ten candidates with the best repayment history.

The proposed definition captures a large class of decision-problems that have been subjected to data-driven approaches. Examples include decisions by banks to grant or deny a loan (Kozodoi et al., 2021), decisions by courts to grant or deny probation (Metz and Satariano, 2020; Završnik, 2021), decisions by public employment agencies to grant or deny participation in active labor market programs (Desiere et al., 2019), and decisions by hospitals to grant or deny certain types of medical treatment (Obermeyer et al., 2019). In each case, an institution has to formulate an allocation principle that regulates which candidates receive a unit of the good and which candidates do not.

### 2.2. Data-driven decision pipeline: Prediction and decision step

How could a solution to the decision problem look like? We already sketched half of the answer: Institutions formulate an allocation principle that regulates how goods are allocated across the candidates. This is the *decision step* in the decision pipeline: The institution uses the decision criterion to select the candidates who receive the good. We implicitly assumed that the decision criterion—the input to the allocation principle—is observed by the institution at the time point of the decision. This is not always the case, however. The decision criterion might be unobserved at decision time because it materializes only in the future or because it is too costly for the institution to measure it for each candidate.

Indeed, many instance of data-driven decision-making are motivated by the fact that the decision criterion is unobserved (or even unobservable) at decision time. Courts would like to base their decision to grant or deny probation on the knowledge about the future criminal behavior of the defendant. Banks would like to base their decisions to grant or deny loans on the knowledge about the future repayment behavior of the loan applicant. Public employment agencies would like to base decisions to grant or deny access to support programs on the knowledge about whether the job-seeker would find re-employment without further support. In all these cases, the decision criterion (criminal behavior, repayment behavior, re-employment) lies in the future and, hence, is unobservable at decision time.

If (and only if) the decision criterion is unobserved at decision time, the decision pipeline is extended by a *prediction step*. In the prediction step, the institution uses observed attributes of the candidate to predict the unobserved decision criterion. Banks would, for instance, use the past repayment history of the candidate (observed attribute) to predict the probability that the candidate will repay the next loan (decision criterion).

**Figure 1:**
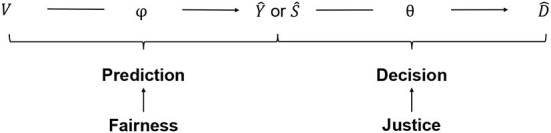
Data-driven decision pipeline.

The resulting two-step decision pipeline is shown in Figure 1. First, the decision criterion is predicted from the observed candidate attributes (prediction step). Then, an allocation decision is made based on the predicted criterion (decision step; Loi et al., 2021; Mitchell et al., 2021).

#### 2.2.1. Decision step

The task of the institution is to find an allocation principle that defines how to select the subset of candidates who receive the good. Let *y*_*i*_ denote the value of the decision-relevant criterion for the i-th candidate. The criterion can be categorical or continuous^3^. Let *d*_*i*_ ∈ {0, 1} be the allocation decision that records whether the *i*-th candidate receives the good (*d*_*i*_ = 1) or not (*d*_*i*_ = 0). Let *Y* and *D* be random variables of the values for a candidate randomly drawn from the population of candidates. The allocation principle is a function θ:*Y* → *D* that maps the decision-relevant criterion *Y* onto the allocation decision *D*. Applying the allocation principle θ(*y*_*i*_) = *d*_*i*_ gives the decision for the *i*-th individual with value *y*_*i*_ on the decision criterion. In words, the allocation principle states: Allocate the good to the *i*-th candidate if and only if the candidate's value on the decision criterion qualifies her for the good. If the decision criterion is not observed at decision time, $\theta \left({ŷ}_{i}\right)={\hat{d}}_{i}$ gives the decision for the *i*-th candidate given their predicted value ŷ_*i*_ on the decision criterion. Ŷ and $\hat{D}$ are the predicted criterion and the prediction-based allocation decision for a candidate randomly drawn from the population.

#### 2.2.2. Prediction step

The prediction step is a classification problem for categorical and a regression problem for continuous decision criteria. The prediction task makes use of a training set of *j* = 1, …, *m* candidates for which the criterion is observed. Let *v*_*j*._ denote the values of the observed features for the *j*-th candidate. We denote additional features that are unobserved, but potentially relevant, as *u*_*j*._. Let *V* and *U* be random variables for the features of a candidate. The prediction task is: Given a training data set of candidates of the form {(*v*_1._, *y*_1_), …, (*v*_*m*._, *y*_*m*_)}, find a function ϕ:*V* → *Y* that maps the observed features onto the criterion. The function ϕ that is estimated in the training data set is then used to obtain predictions of the criterion value for the candidates of interest at decision time. For a continuous decision criterion, ϕ(*v*_*i*._) = ŷ_*i*_ returns the predicted value of the *i*-th candidate's criterion value. Candidates can be ranked according to their predicted value of the decision criterion. For a categorical decision criterion, $\phi \left(v_{i.}\right)={ŝ}_{i}=\hat{P}\left(y_{i}=1\right)$ returns the score ŝ_*i*_, the predicted probability that the *i*-th candidate possesses the decision criterion. The scores can be used to rank candidates according to their predicted probability of possessing the criterion. Let Ŝ be the score for a random candidate.

### 2.3. Algorithmic fairness and justice

The remainder of the paper explicates the implications of a consequent distinction between prediction and decision for the design and evaluation of data-driven decision procedures. *Our main argument is that the distinction between prediction and decision implies a corresponding distinction between the concepts of fairness and justice*. We propose that fairness is a property of the prediction algorithm and is only relevant at the prediction step. Justice is a property of the allocation principle and only relevant at the decision step. We propose the following definitions of justice and fairness in the context of data-driven decision-making.

** Definition 1 (algorithmic justice)**. An allocation principle is called just iff it approximates a well-justified distributive justice principle.

** Definition 2 (algorithmic fairness)**. A prediction algorithm is called fair iff its predictions satisfy a well-justified substantive fairness definition.

The definition of algorithmic justice is elaborated in Section 3. Working within the framework of local justice (Elster, 1992), we show how the design of allocation principles can be guided by middle-range justice principles. The definition of algorithmic fairness is further discussed in Section 4. At this point, we note that our definition of algorithmic fairness is kept at a very general level and, by design, can accommodate a large range of substantive fairness definitions that have been proposed in the literature (Mitchell et al., 2021). Substantive fairness definitions formally describe the concrete properties that algorithmic predictions must satisfy in order to be considered fair. Independence (also called Statistical Parity), for instance, requires that predictions are statistically independent from protected attributes like gender and ethnicity.

Note that justice and fairness are indeed separate concepts. A just allocation principle does not guarantee a fair prediction algorithm and vice versa. For instance, the final outcome of the data-driven decision process—the decision to allocate the good to a candidate or not—can be just but unfair. The decision that a candidate does not receive a loan might be just because the bank's allocation principle to choose the candidates with the highest predicted probability to repay approximates the well-justified desert-based justice principle. At the same time, the (prediction-based) decision might be unfair because the algorithm that predicts the repayment probability systematically under-predicts the repayment probability of women compared to men. In the same vein, the outcome of the data-driven decision process might be fair but unjust. It becomes obvious that we need both: Data-driven decision-making should be fair and just. Importantly, there is no conflict between fairness and justice. We actually can have both and do not need to trade off one against the other.

## 3. Just decisions

### 3.1. Local justice

Local justice is concerned with the allocation of goods to individuals by relatively autonomous meso-level institutions (Elster, 1992; Schmidt, 1992b). Institutions are formal organizations that, in fulfilling their respective function, make decisions about the allocation of goods (Schmidt, 1992a). Local justice problems are local in the double sense that (a) they are solved de-centrally by relatively autonomous institutions and (b) their solutions are highly context-dependent and vary across sectors or “localities” within one society. Global justice, in contrast, is concerned with the overall design of the basic structure of society (Rawls, 1971), the “constitutional ground rules of a social, political, and economic order” (Schmidt, 1994, 322). The class of decision problems discussed in this paper clearly falls within the scope of local justice.

The building blocks of local justice are (a) the *good* that is allocated, (b) the individuals (*candidates*) to whom the good can potentially be allocated, (c) some functional rule (*allocation principle*) that specifies how goods are allocated to candidates, and (d) a normative standard (*distributive justice principle*) against which the resulting allocation is evaluated (Cohen, 1987). Goods, candidates, and allocation principles were already introduced as part of the decision problem in Section 2.1. The following discussion, therefore, focuses on the distributive justice principles and how they can guide the selection of allocation principles.

#### 3.1.1. Distributive justice principles

Distributive justice principles are well-justified accounts of how goods should be allocated. The justice principles define an ideal standard against which non-ideal allocation principles—that have to operate under non-ideal real-world conditions—are evaluated. Generally, we wish to select the allocation principle or combination of allocation principles that best approximates our preferred distributive justice principle. Our focus lies on what we call *middle-range distributive justice principles*. Middle-range principles are general enough to apply across multiple empirical cases. At the same time, they are not as general as global justice theories that aim to regulate the basic structure of society but give little guidance for the resolution of concrete allocation problems. Examples of global justice theory include *A Theory of Justice* (Rawls, 1971), *Anarchy, State, and Utopia* (Nozick, 1974), and the hypothetical insurance scheme laid out by *Luck Egalitarianism* (Dworkin, 1981).

In the next section, we discuss four middle-range distributive justice principles: equality, desert, need, and efficiency (Deutsch, 1975; Konow, 2003; Törnblom and Kazemi, 2015). The principles draw inspiration from broader distributive justice theories, namely egalitarianism (Arneson, 2013), desert-based justice (Feldman and Skow, 2020), sufficiency (Brock, 2018) and prioritarianism (Parfit, 1997; Adler and Holtug, 2019), and consequentialism (Sinnott-Armstrong, 2021), respectively.

Distributive justice principles are context-dependent, pluralistic, and contested (Schmidt, 1994; Konow, 2003). *Context dependency* means that the selection of justice principles is guided and justified by the empirical facts that characterize the concrete allocation problem. No distributive justice principle satisfies justified moral expectations in every empirical case. *Pluralism* emphasizes that there are allocation problems for which multiple (potentially conflicting) justice principles are equally well-justified such that a compromise between principles is necessary. The *contestedness* of justice principles highlights that the allocating institution is often subject to demands other than justice, such as profitability or public preferences, that preclude the implementation of the preferred justice principle.

#### 3.1.2. From justice principle to allocation principle

The process of formulating an allocation principle is akin to the operationalization of a latent construct for empirical research. Each middle-range distributive justice principle identifies a distinct latent construct—equality, desert, need, or efficiency—that should guide the allocation of goods. Formulating an allocation principle amounts to finding a manifest indicator for this latent construct. The indicator is a context-fitting interpretation of the justice principle in the sense that it transports the general intention of the justice principle into the specific allocation context. For instance, life expectancy might be a manifest indicator for the latent concept *need* in the context of allocating kidney transplants. Repayment probability might be a manifest indicator for the latent concept *desert* in the context of allocating loans.

#### 3.1.3. Choosing allocation principles

Local justice is a descriptive (and partly explanatory) rather than normative approach (Elster, 1992). Local justice has three broad goals: (1) Cataloging the allocation principles implemented by existing institutions. (2) Identifying the mechanisms that lead to the implementation of certain types of principles in certain types of allocation problems. (3) Describing the typical distributive consequences associated with each principle. The distributive consequences encompass the direct results (how gets what?) and also the indirect (unintended) incentive effects of an allocation principle^4^. Local justice does not provide a normative argument for why a certain principle should be chosen. It does not formulate a moral justification—in the sense of a rational defense of the principle to all candidates who are eventually affected by it—for why a certain principle should be chosen.

In this realist (rather than idealist or normative) perspective, local justice shows that the selection of allocation principles results from complex negotiation and bargaining between the allocating institution, political actors, the candidate population, and the overall public represented by the media (Elster, 1992; Schmidt, 1992a). The actors are (at least partially) aware of the distributive consequences of different allocation principles and formulate their preferences accordingly. Which allocation principle is selected depends on the relative bargaining power of the actors. Classically, bargaining power is a function of actors' relative dependence on each other. The less dependent an actor is on the others for realizing her preferences, the higher her bargaining power. Bargaining is also a discourse, however, in which the best argument may win irrespective of the nominal bargaining power of the actor who formulates the argument. The actors can, therefore, be expected to leverage moral arguments and justifications that support their preferred allocation principle. These arguments and justifications are drawn from the middle-range distributive justice principles. An exact explanation of the negotiation process underlying the selection of allocation principles is not the purpose of the paper. Suggestions for the organization of such negotiation processes are formulated in the literature on impact assessment frameworks for data-driven decision systems (Selbst, 2018; Mantelero, 2018; Metcalf et al., 2021).

We also adopt the realist approach. That is, we do not provide a universal argument for why a certain type of allocation principle should always be selected for a certain type of allocation problem. It might turn out that such a universal argument does not exist. We, at least, are not aware of such an argument. Instead, we sketch the likely distributive consequences of each principle and present the main arguments that justify the implementation of the principle. The ultimate selection of an allocation principle is the task of the actors who are embedded in the allocation context. Our hope is that a better understanding of the allocation principles helps these actors to make better decisions.

### 3.2. Distributive justice principles

Four middle-range distributive justice principles are discussed: equality (E), desert (D), need (N), and efficiency (EFF) (Deutsch, 1975; Konow, 2003; Törnblom and Kazemi, 2015). Table 1 provides a short definition of each principle. Note that sub-forms of the main justice principles exist (Törnblom and Kazemi, 2015). While we try to present a comprehensive list of justice principles and their sub-forms, we do not claim that our list is exhaustive. The principles defined below are our interpretations of the underlying middle-range distributive justice theories. Other interpretations are certainly possible and may prove to better reflect the central concerns of the underlying theories. For now, however, our definitions should provide a useful starting point.

**Table 1:** Middle-range distributive justice principles.

**Justice principle**	**Sub-form**	**Decision criterion**	**Allocation principle**
Equality (E)	Equality of treatment (Et)	Selection by lottery	Allocate the good to the candidate if and only if the candidate is selected by an unbiased lottery.
	Equality of outcome (Eo)	Post-allocation outcome	Allocate the good to the candidate if and only if this allocation is part of an overall allocation scheme that minimizes inequality in the distribution of post-allocation outcomes.
Desert(D)	Productive contribution (Dp)	Past or future contribution to cooperative production of the good	Rank candidates according to their desert in descending order. Allocate the good to the candidate if and only if the candidate is among the top-ranked candidates.
	Effort (De)	Effort expended in the cooperative production of the good	
	Costs and Sacrifice (Dc)	Costs incurred in the cooperative production of the good	
Need (N)	Biological (Nbi)	Need for goods essential for survival	Rank candidates according to their need in descending order. Allocate the good to the candidate if and only if the candidate is among the top-ranked candidates.
	Basic (Nba)	Need for goods essential for recognizably human life	
	Functional (Nf)	Need for goods essential for fulfilling social roles	
Efficiency (EFF)	–	Outcome-increment realized by allocating good to candidate	Rank candidates according to their outcome-increment in descending order. Allocate the good to the candidate if and only if the candidate is among the top-ranked candidates.

#### 3.2.1. Equality

The equality (E) principle requires either equal treatment or equal outcomes across candidates. Equal treatment (Et) demands that all candidates receive the same amount of the good. When the good is scarce and indivisible, it is impossible to implement this principle. Lotteries are a second-best approximation in this case: Each candidate has the same probability *p* = *X*/*n* to receive the good (Elster, 1992). The decision criterion *Y* is the selection result of the lottery. The corresponding allocation principle θ is: Allocate the good to the *i*-th candidate if and only if the candidate is selected by the lottery.

Equal outcomes (Eo) demands that candidates have the same post-allocation outcome, i.e., the same outcome after the allocation decision is implemented. This raises the thorny problems of (a) defining the relevant outcome, (b) estimating the—potentially inter-individually varying—effect of the good on the outcome, and (c) defining a metric that measures inequality in outcomes. The decision criterion *Y* is the post-allocation outcome. The corresponding allocation principle θ is: Allocate the good to the *i*-th candidate if and only if this allocation is part of an overall allocation scheme that minimizes inequality (as measured by the metric) in the distribution of post-allocation outcomes across candidates^5^. If multiple allocation schemes minimize inequality to the same extent, a rule must be defined to select one of the schemes (e.g., a random draw).

**Figure 2:**
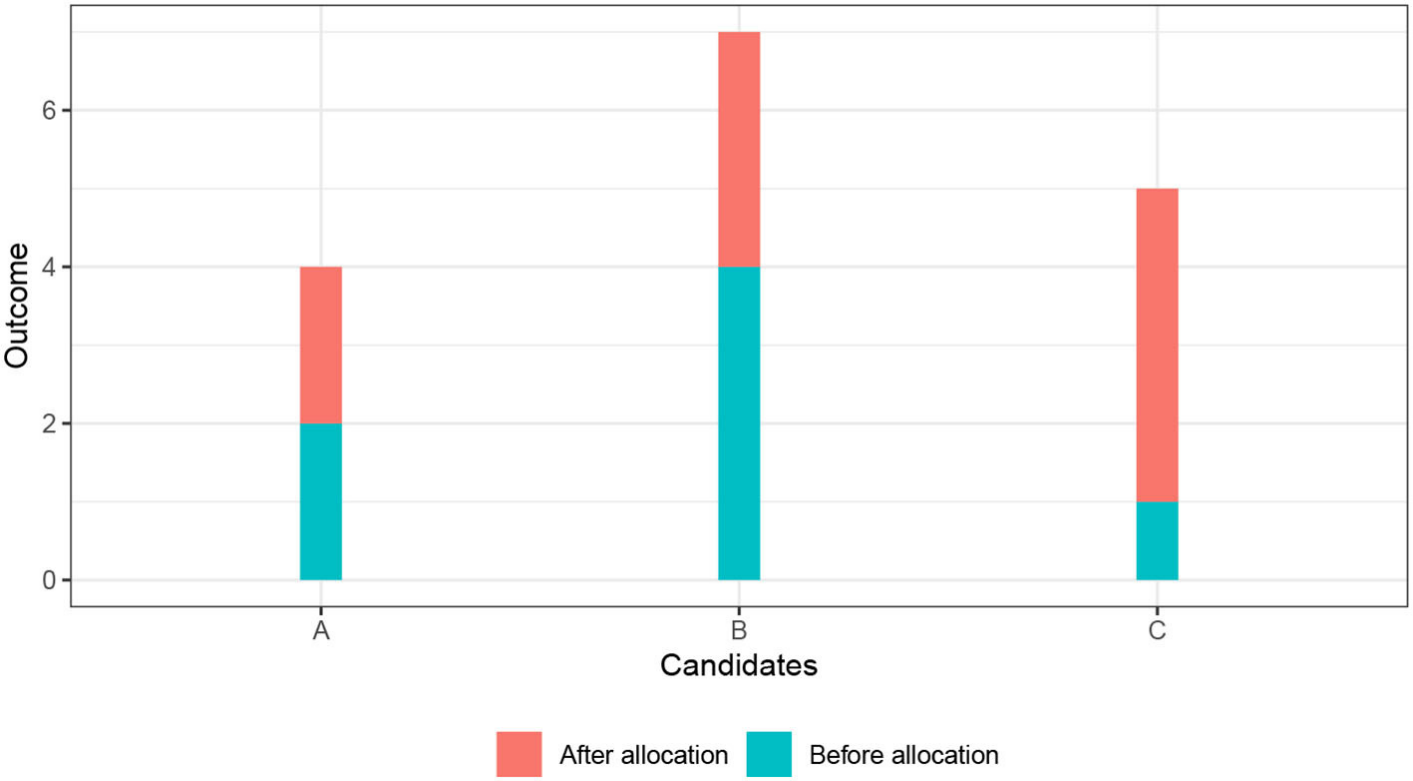
Illustration of equal outcomes principle.

Figure 2 illustrates the equal outcome (Eo) principle. There are three candidates (A, B, and C), shown on the X-axis. The Y-axis shows the outcomes of the candidates. The height of the blue bar indicates the outcome of the candidate before the allocation decision is made. The height of the orange bar indicates the increase in the outcome that the candidate would experience if the good is allocated to her. The impact of the good on the outcome differs across the candidates. The outcome of candidate A would increase by the smallest amount, the outcome of candidate C by the largest amount. The combined height of the orange bar and the blue bar indicates the level of the outcome after the allocation—under the condition that the candidate receives the good. Imagine that there are *X* = 2 units of the good that we can allocate to the three candidates. Because we do not want to be wasteful (and to avoid the leveling-down objection), we allocate both units of the good, even if a more equal state could be reached if we allocate fewer units of the good^6^. There are three possibilities to allocate the two goods to the three candidates: (1) Allocate to A and B, (2) allocate to A and C, and (3) allocate to B and C. Following the equal outcome (Eo) principle, we choose the allocation scheme that minimizes inequality in the post-allocation outcomes. We measure inequality with the Gini-coefficient. The Gini-coefficient varies between 0 (perfect equality: every candidate has the same outcome) and 1 (perfect inequality: only one candidate has a positive outcome, the outcome of the other candidates is zero). The Gini-coefficients for the allocation schemes are 0.33, 0.05, and 0.24, respectively. Accordingly, the equal opportunity (Eo) principle recommends allocating the good to candidate A and candidate C.

The equality principle is justified by the egalitarian ideal that all candidates are moral equals—at least with respect to the factors that are morally relevant to the allocation problem and should therefore affect its outcome (Gosepath, 2011; Arneson, 2013). Equality is the baseline principle whenever no candidate can make an inter-personally comprehensible and acceptable claim to more than an equal share. Such claims might refer to personal need and desert or to gains in efficiency realized by allocating the good to a specific candidate. The equality principle likely produces negative incentive effects: Candidates are not held responsible for their actions and—especially in the case of equality of outcomes—can count on compensation for socially harmful actions that lower their pre-allocation outcomes.

#### 3.2.2. Desert

The desert (D) principle ties the allocation of goods to so-called desert-bases (Moriarty, 2018; Feldman and Skow, 2020). Desert-bases are properties of an individual by virtue of which the individual can make a claim to the good. The decision criterion *Y* is the desert-base deemed relevant in the allocation context. The allocation principle θ is: Rank the candidates according to their desert *Y* in descending order. Allocate the good to the *i*-th candidate if and only if the candidate is among the X top-ranked candidates. If desert is unobserved at decision time, candidates are ranked based on either the predicted value Ŷ for continuous desert-bases or the score Ŝ for categorical desert-bases.

Not all properties qualify as desert-bases (Feldman and Skow, 2020). Desert-bases generally reflect socially beneficial properties and actions. The bases should be morally-relevant, i.e., they should stand in some relation to the good. It should be possible to evaluate desert-bases as good or bad. Only then can we say that a candidate has a stronger claim to the benefit (burden) because she has a property or performed an action that is deemed good (bad). Desert-bases can be limited to properties and actions for which the candidate can be reasonably held responsible (Arneson, 2015). Candidates cannot be held responsible for things that are not under their control (Lippert-Rasmussen, 2018) or that result from brute luck (Dworkin, 1981), i.e., from outcomes of gambles that candidates could not anticipate or could not decline because they lacked a reasonable alternative.

In contexts concerned with the allocation of goods produced *via* cooperation, three desert-bases are often proposed: past or future contribution of the individual to the production of the good (Dp), effort expended in the production process (De), and costs or sacrifices incurred due to the production activity (Dc) (Lamont and Favor, 2017).

**Figure 3:**
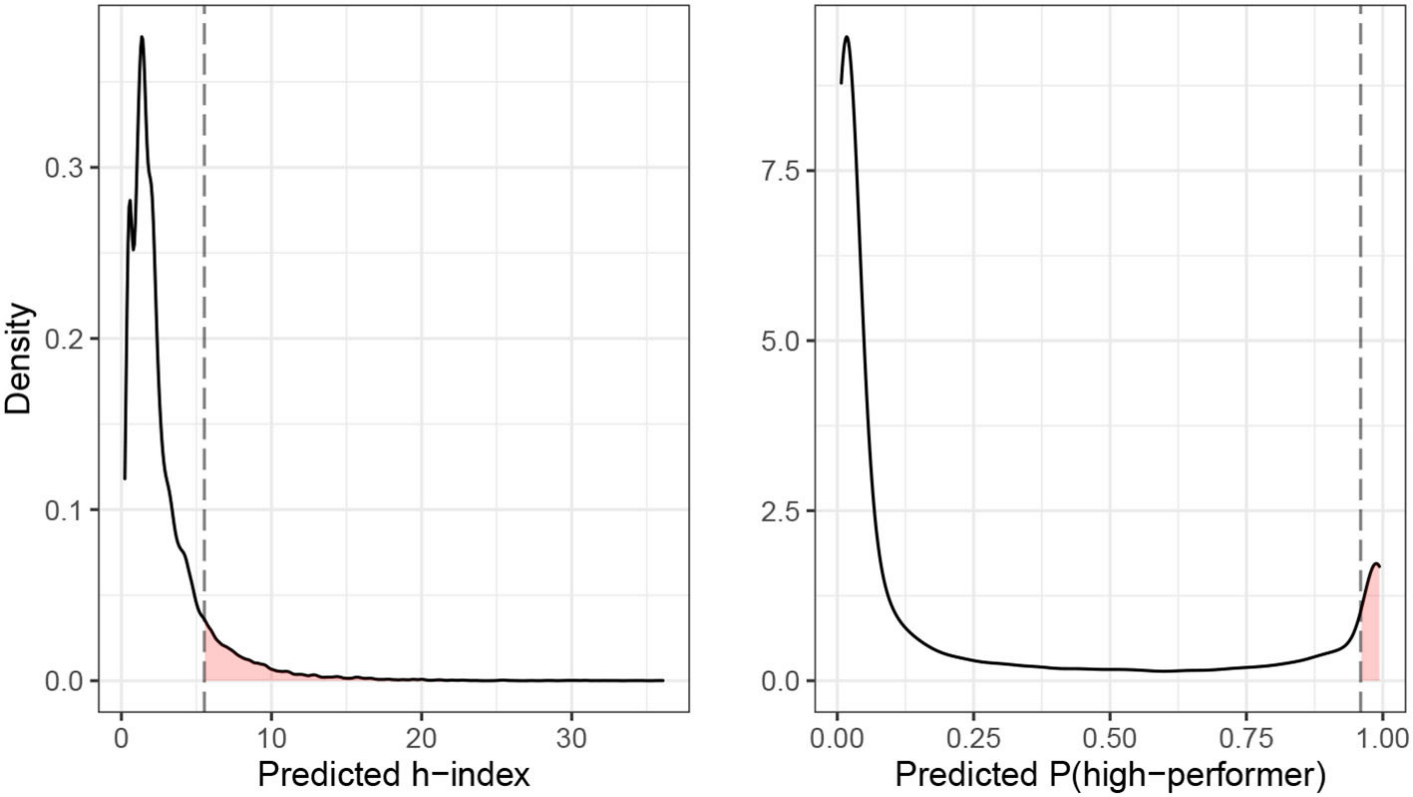
Illustration of desert principle.

Figure 3 illustrates the desert principle. Consider a university department that wants to allocate job-interviews for open tenure-track positions (the good) based on desert (latent decision criterion). Here, desert is operationalized as the future h-index (manifest decision criterion) of a candidate. The h-index quantifies the scientific impact of a researcher based on her number publications and the number of citations that her publications received (Hirsch, 2005). An h-index of k indicates that the k most cited papers of a researcher received at least k citations. The university department wants to invite 10% of the candidates to job-interviews. We consider two scenarios: (1) The department invites candidates whose predicted h-index is in the top-10% of the candidate distribution. (2) The department invites candidates whose predicted probability to become a high-performer is among the top-10% of the candidate distribution. High-performers are candidates whose predicted h-index is above the 75% percentile of the candidate distribution. The first scenario describes a regression problem and the allocation decision is based on the predicted value Ŷ of the h-index. The second scenario describes a classification problem (candidate is either a high-performer or not) and the allocation decision is based on the predicted score Ŝ of becoming a high-performer. The left panel of Figure 3 shows the density plot for the h-index prediction, the right panel shows the density plot for the high-performer prediction^7^. In both cases, the candidates whose predicted value (either Ŷ or Ŝ) falls in the red area to the right of the dashed line are invited to the job-interview. According to the chosen indicator of desert, these are the candidates with the strongest claim to the good.

The desert principle is justified whenever reasonable desert-bases exist and are not overridden by other concerns like need or efficiency. Then, candidates can make an inter-personally comprehensible and acceptable claim to more than an equal share of the good that is based on their personal desert. The egalitarian ideal of the candidates' moral equality does not prescribe equality *per se* (Gosepath, 2011). Treating candidates as moral equals can also mean to take their actions and responsibility serious and to allocate goods accordingly (Moriarty, 2018). The desert principle can produce positive incentive effects: Candidates are rewarded for socially productive behavior and punished for harmful behavior. It can be difficult, however, to identify desert-bases for which candidates can be truly held responsible.

#### 3.2.3. Need

The need (N) principle ties the allocation of goods to need claims (Brock, 2018). Need claims have the following structure: The candidate requires the good in order to realize a certain end-state. Following prioritarianism (Parfit, 1997; Holtug, 2007), the strength of a need claim to the good increases the worse-off the candidate is, i.e., the farther away the candidate is from achieving the end-state. The decision criterion *Y* is the strength of the need claim. Need claims grow in strength the farther away the candidate is from the end-state prior to the allocation. The allocation principle θ is: Rank the candidates according to the strength of their need claim *Y*. Allocate the good to the *i*-th candidate if and only if the candidate is among the X top-ranked candidates. If need is unobserved at decision time, candidates are ranked based on either predicted value Ŷ for continuous end-states or the score Ŝ for categorical end-states.

Three classes of end-states generally qualify as bases for need claims (Törnblom and Kazemi, 2015; Brock, 2018): Biological needs (Nbi) are states that are essential to survival. Basic needs (Nba) are states that are essential to lead a recognizably human life, according to the standards of one's society. Functional needs (Nf) are states that enable the candidates to fulfill their social roles. Need justifies claims to the good irrespective of whether the candidate is responsible or not for failing to achieve the end-state.

Going back to the university department example in Figure 3, we could imagine that the department wants to allocate a career support program (the good) to its current employees. The department decides to allocate the program based on need, operationalized as the predicted h-index of the employees. The department could allocate the program to the 10% of employees with the lowest predicted h-index (Ŷ) or to the 10% of employees with the lowest predicted probability of becoming a high-performer (Ŝ). The idea is that a high h-index (continuous end-state) and being a high-performer (categorical end-state) are valuable end-states for researchers and that the support program helps researchers to realize these end-state. Employees with a low h-index are farther away from realizing the end-state and, therefore, have a stronger need claim to the support program.

The need principle is justified whenever reasonable need claims exist and are not overridden by other concerns like desert or efficiency. Then, candidates can make an inter-personally comprehensible and acceptable claim to more than an equal share of the good that is based on their personal need. Treating all candidates as moral equals (Gosepath, 2011) does not necessarily mean to equalize outcomes but can also mean to work toward a situation in which all candidates can at least fulfill their biological, basic, and functional needs (Brock, 2018). Meeting needs is socially beneficial as it enables the candidates to function as productive members of society. The need principle can produce negative incentive effects: Candidates are not held responsible for their need and, hence, are not punished if they squander the allocated good because they expect additional transfers in the future.

#### 3.2.4. Efficiency

The efficiency (EFF) principle allocates goods to promote a valued outcome (Elster, 1992). Goods are allocated across candidates in a way that maximizes the degree to which the outcome is attained in the aggregate^8^. The decision criterion *Y* is the increment in outcome-attainment that is realized by allocating the good to a specific candidate. In other words: The criterion is the candidate-specific effect of the good on the outcome. The allocation principle θ is: Rank, in descending order, the candidates according to the increment in outcome-attainment *Y* that is achieved by allocating the good to the candidate. Allocate the good to the *i*-th candidate if and only if the candidate is among the X-top ranked candidates^9^.

Figure 2 that we used to illustrate the equal outcome principle can also illustrate the efficiency principle. Remember that the effect of the good on the outcome (the height of the orange bar) differed across the three candidates A, B, and C. The effect was strongest for candidate C (four outcome units), followed by candidate B (three outcomes units), and then candidate A (two outcome units). The efficiency principle recommends to allocate the two units of the good to candidate B and candidate C. This happens to be a much more unequal allocation (Gini-coefficient of 0.24) then the one recommended by the equal outcome principle (Gini-coefficient of 0.05). It is, however, not generally true that the efficiency principle necessarily favors unequal allocations.

Concerns for efficiency arise whenever the effect of the good on the outcome differs across candidates. Then, candidates can make an inter-personally comprehensive and acceptable claim to more than an equal share of the good that is based on the gain in efficiency that is realized by allocating the good to them. Concerns for equality, desert, and need can override efficiency claims, however—especially because efficient allocations can be very unequal and might not benefit the candidates with the highest desert or need. The efficiency principle, as formulated here, is a local version of consequentialism (Sinnott-Armstrong, 2021)^10^. It is justified by a concern for maximizing the aggregate well-being (in terms of outcome attainment) of the candidate pool, the allocating institution, or society as a whole. The principle likely produces no incentive effects because candidates cannot actively influence the size of the outcome increment that is gained by allocating the good to them.

### 3.3. Combining principles

Due to the pluralism of distributive justice principles, there are frequently allocation problems for which multiple (potentially conflicting) principles are equally well-justified. Strategies for building compromises between principles include: (a) Combining decision criteria *via* a *weighting* scheme (Konow, 2003). Each of the C different decision criteria *Y*_*c*_ is assigned a weight *w*_*c*_ and the allocation decision is based on the weighted sum $Y^{*}=\overset{C}{\underset{c=1}{\sum }}w_{c}\cdot Y_{c}$ of the criteria. (b) Establishing a hierarchical ordering of the principles, where higher-ordered principles take precedence and lower-ordered principles break ties (Törnblom and Kazemi, 2015). (c) Conjunctive (disjunctive) procedures, where candidates are ranked according to the decision criterion on which they score lowest (highest) (Elster, 1992). The allocation decision is then based on the combined rank order.

Note that a given decision criterion can also be over-determined, i.e., supported by multiple justice principles. For instance, in a medical context, need and efficiency suggest allocating a kidney transplant to the candidate with the lowest pre-allocation life expectancy because (a) this expresses a concern for the worst-off and (b) the gain in additional life expectancy is highest for this candidate. Over-determination facilitates the selection of an allocation principle because it is easier to build a winning coalition of actors who support the principle (Elster, 1992).

## 4. Fair predictions

In Section 2.3, we introduced a general definition of algorithmic fairness and noted that it is compatible with a large range of substantive fairness metrics. It is beyond the scope of this paper to comprehensively review existing fairness metrics. Interested readers are referred to the summary article of Mitchell et al. (2021). Let us highlight, however, that many of the most popular metrics share a focus on equalizing predictions (independence, counterfactual fairness) or prediction errors (equal accuracy, sufficiency, separation) across groups that are defined by so-called protected attributes drawn from anti-discrimination law. Protected attributes include, amongst others, sex, gender, sexuality, ethnicity and race, and disability (Barocas and Selbst, 2016). Inequalities in predictions or prediction errors that are not systematically associated with these protected attributes are not considered as relevant instances of unfairness. It has been shown that these metrics are motivated by moral arguments derived from equality of opportunity theories (Heidari et al., 2019; Castro et al., 2021; Loi et al., 2021). Equality of opportunity, mostly applied in the allocation of social positions, states that access to goods (e.g., a job position) should only depend on candidates' qualification for the good (e.g., their educational credentials) and not on any other (morally arbitrary) attributes of the candidates (Arneson, 2015).

We argue that the exclusive focus on protected attributes is too narrow for data-driven decision procedures, an argument to which we return in Section 5. Our main point is the following: The exclusive focus on protected attributes is not justified because any systematic tendency of the prediction algorithm to assign more prediction error to a group—protected or unprotected—is unfair. Each systematically biased prediction algorithm creates a new algorithm-specific group of candidates who are systematically disadvantaged and have a reasonable claim to protection (Fazelpour and Lipton, 2020). Further, equality of opportunity is intended to regulate the allocation of goods, not the allocation of prediction errors. It cannot, therefore, be used to justify a certain allocation of prediction errors. It might be justified to account for protected attributes at the decision step. However, the same is not true at the prediction step because all candidates, irrespective of their membership in protected groups, have the same claim to receiving equally good predictions.

In an attempt to go beyond the narrow focus on protected attributes, we now provide a formal representation of our notion of algorithmic fairness, which we call *error fairness*.

** Definition 3 (error fairness)**. Let V be the observed features and U be the unobserved features of the candidates. Let ε measure the deviation between the predicted criterion value (either Ŷ or Ŝ) and the observed criterion value *Y*. A prediction algorithm is error-fair iff ε ⊥ (*V, U*).

For continuous decision criteria, the residuals (ŷ_*i*_ − *y*_*i*_) may be used as an error measure. For binary criteria, pseudo-residuals (ŝ_*i*_ − *y*_*i*_) may be used to measure deviations. However, our notion of error fairness is not tied to a specific error type and other measures could be considered dependent on the application context.

Error fairness is satisfied if prediction errors are not systematically related to (i.e., statistically independent of) observed or unobserved candidate features. An error-fair prediction algorithm accomplishes the prediction task equally well for all candidates without systematic error. We acknowledge two caveats of error fairness: (a) It is impossible to check the statistical independence between unobserved candidate features and prediction errors. (b) It is very difficult or even impossible to satisfy error fairness perfectly. Nevertheless, we maintain that error fairness is valuable as an aspirational goal. It motivates us to check, within our capabilities, whether prediction errors systematically befall certain segments of the candidate population. We return to this point in Section 5, where error fairness is embedded into a broader discussion of bias in data-driven decision-making.

With these limitations in mind, an approach for measuring the degree to which a prediction algorithm satisfies error fairness could proceed as follows. Error fairness is assessed in an independent test sample of candidates who were not used for model training. Prediction errors are computed using a task-specific error measure (e.g., pseudo-residuals for classification tasks). A linear regression of the prediction errors on the observed candidate features *V* (including interactions between candidate features and non-linear terms) is performed. More flexible types of regression methods could be considered to capture complex relationships between *V* and ε. The *R*^2^ ∈ [0, 1] statistic of the regression model—the share of variance in the errors explained by the observed features—is the fairness metric. Large *R*^2^ indicate that there are systematic relationships between observed candidate features and the prediction errors. The larger the *R*^2^, the more the prediction algorithm violates error fairness. The underlying idea of the *R*^2^-metric is similar in spirit to the first step of multi-accuracy boosting (Kim et al., 2019), which aims to identify subgroups of candidates for which a prediction algorithm produces large prediction error. In addition, mutual information (Cover and Thomas, 2006, chapter 2), which is not limited to linear dependence, could be used as an alternative metric to check independence of prediction errors from observed candidate features^11^.

These approaches, however, can only check the independence of prediction errors from observed candidate features *V* and not from unobserved candidate features *U*. Even if the algorithm has a small *R*^2^, it may violate error fairness due to dependence of deviations on unobserved features—a small *R*^2^ is a necessary but not sufficient condition for error fairness.

## 5. Pitfalls and biases of data-driven decision-making

Existing research on data-driven decision-making identified a series of biases that can affect data-driven decisions and lead to systematic discrimination against segments of the candidate population (Mehrabi et al., 2019; Suresh and Guttag, 2020). Here, we extend the framework of Friedler et al. (2021) to explicitly account for the distinction of prediction and decision step. We thereby also illustrate why the notion of error fairness is a valuable aspirational goal, even if it will be difficult or even impossible to achieve it perfectly in real-world applications.

Building on Friedler et al. (2021), we distinguish four spaces: The construct space (CS) contains the latent decision criteria identified by the middle-range distributive justice principles—namely equality, desert, need, and efficiency. The indicator space (IS) contains the manifest decision criteria that are chosen to operationalize the latent criteria. Construct space and indicator space are connected by the operation of operationalization. The measurement space (MS) contains the measured values of the chosen manifest decision criterion. These are the *Y*s in our notation. Indicator space and measurement space are connected by the operation of measurement. Finally, the prediction space (PS) contains the predicted values of the decision criterion. These are the Ŷ (for continuous decision criteria) and Ŝ (for categorical decision criteria) in our notation. Measurement space and prediction space are connected by the operation of prediction. The prediction space is only needed if the decision criterion is unobserved at decision time. In this case, the measured decision criterion is only available in the training data, not for the candidates for which an allocation decision must be made^12^.

**Figure 4:**
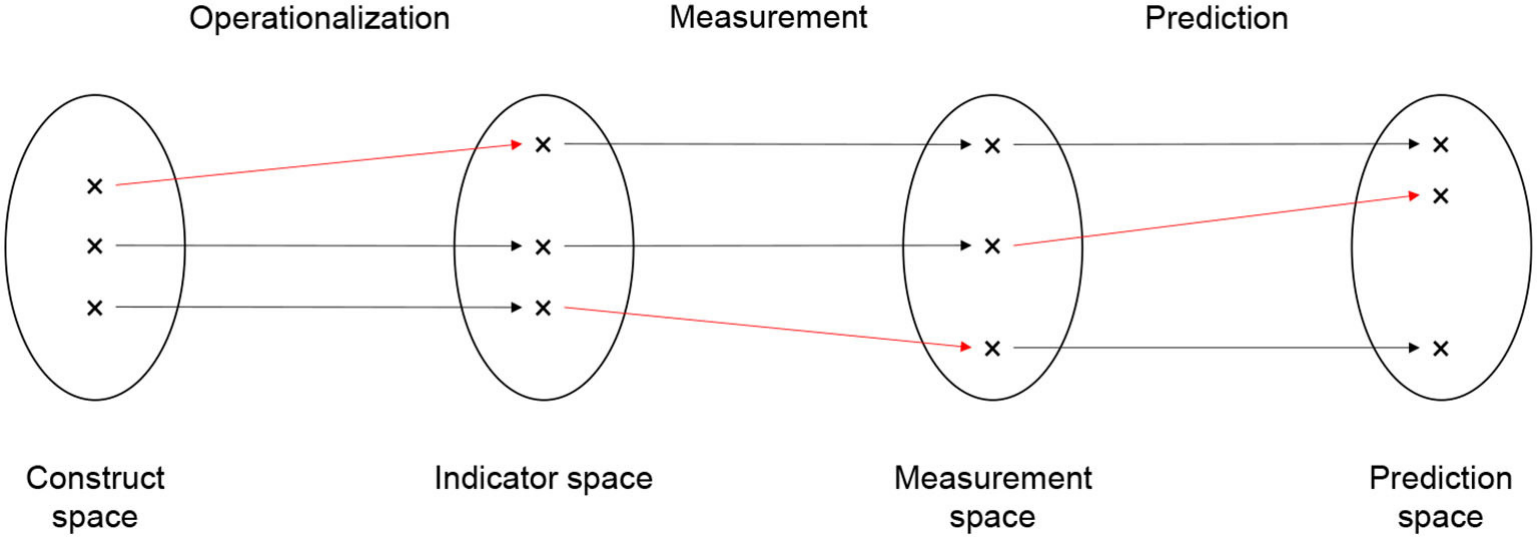
Spaces and biases.

Figure 4 depicts the four spaces (construct, indicator, measurement, and prediction space) as circles and the three operations that connect the spaces (operationalization, measurement, and prediction) as arrows. There are three candidates whose relative positions in the spaces are indicated by the black crosses. The crosses that are connected by arrows all belong to the same candidate. The relative position of a candidate indicates her value on the latent decision criterion (in the construct space), the manifest decision criterion (in the indicator space), the measured decision criterion (in the measurement space), and the predicted decision criterion (in the measurement space) in relation to the values of the other candidates.

Bias can arise at the transition from one space to the next, that is, in the operations we called operationalization, measurement, and prediction. Bias is present if the operations change the relative distance between candidates in the spaces. Accordingly, there are three types of bias: operationalization bias, measurement bias, and prediction bias. In Figure 4, the biases are indicated by red arrows. The red arrows are not strictly horizontal, indicating that the relative distance between the candidate changes. Starting from the left, the first red arrow indicates operationalization bias, the second red arrow indicates measurement bias, and the third red arrow indicates prediction bias.

*Operationalization bias* is present if the relative distance between candidates on the manifest decision criterion differs from their distance on the latent construct. Consider, for instance, a hospital that wants to allocate access to treatment options (the good) based on patients medical need (the latent decision criterion; Obermeyer et al., 2019). Patients with higher needs should receive more treatment. Medical need is operationalized as a patient's past spending on treatment (manifest decision criterion) under the assumption that patients with higher needs will have spend more money on treatment. Bias is introduced because poor patients cannot spend as much on treatment as wealthier patients, even if they have the same level of medical need. The operationalization under-represents the medical need of poor patients but not the medical need of wealthy patients. Accordingly, the distance between poor and wealthy patients in the indicator space will be larger than their distance in the construct space.

*Measurement bias* is present if the relative distance between candidates on the measured decision criterion differs from their distance on the manifest decision criterion. Consider a probation panel that wants to allocate probation (the good) based on a convicted person's desert (the latent decision criterion). Desert is operationalized as the number of re-offenses of the convicted person in the past (the manifest decision criterion). The number of re-offenses is measured as the number of re-arrests (the measured decision criterion) of that person, as recorded in police documents. In the US (and probably also in other countries), the number of re-arrests is a biased measure of the number re-offenses (Lum and Isaac, 2016). Black persons, for instance, are more likely to be arrested than White persons even if they re-offend to the same level. The measurement under-represents the number of re-offenses among White persons but not among Black persons. Accordingly, the distance between Black persons and White persons in the measurement space is larger than their distance in the indicator space.

*Prediction bias* is present if the relative distance between candidates on the predicted decision criterion differs from their distance on the measured decision criterion. Prediction bias occurs for a number of reasons (Mehrabi et al., 2019; Suresh and Guttag, 2020). It arises, for instance, when the data generating process changes such that the training data on which the prediction model is estimated were generated by a different process than the data for the candidates to which the estimated model is eventually applied. Consider a public employment agency that wants to allocate access to support programs (the good) based on need (the latent decision criterion), which is operationalized by re-employment after a maximum of 6 months of job-search (manifest decision criterion) and measured without bias. Consider further that the training data were generated by a process that features discrimination against female job-seekers. That is, female job-seekers in the training data are less likely than male job-seekers to find re-employment. The prediction model learns the association between gender and re-employment and, therefore, predicts a lower re-employment probability for female job-seekers than for male job-seekers. Imagine (somewhat unrealistically) that gender discrimination would suddenly disappear from one day to the next. Female job-seekers in the candidate pool would no longer be less likely to find re-employment than male job-seekers. Bias is introduced because the prediction model continues to predict lower re-employment probabilities for female than for male job-seekers (unless it is retrained on newer observations). The predictions under-represent the re-employment probability among female job-seekers but not among male job-seekers. Accordingly, the distance between female job-seekers and male job-seekers in the prediction space is larger than their distance in the measurement space.

Bias that is introduced at one transition tends to be carried forward to later transitions, unless there is a purposeful de-biasing or different biases happen to cancel each other out. Consider again the case of the hospital that wants to allocate medical treatment based on medical need. Due to the operationalization bias, the distance between poor and wealthy patients on medical spending (manifest decision criterion) is larger than the difference on medical need (latent decision criterion). Even if medical spending is measured without bias, the difference on measured medical spending (measured decision criterion) between poor and wealthy patients is larger than the difference on medical need (latent decision criterion). The lesson is: It is necessary to think about all three types of bias. The absence of prediction bias, for instance, does not guarantee that there is no measurement bias or operationalization bias. The major problem is that we usually do not know the relative distance between candidates' latent decision criteria in the construct space. If we did, we would not need to go through the entire process of operationalizing, measuring, and (sometimes) predicting. Similarly, we usually do not know the relative distance between candidates' manifest decision criteria in the indicator space. The distances become only visible after we applied the measurement operation. Substantive background knowledge and critical thinking appear to be the most effective weapons to detect operationalization bias and measurement bias. The same is true for prediction bias, with the addition that we can rely on fairness metrics (Mitchell et al., 2021) to detect bias.

In the absence of these three biases, it is reasonable to assume that the measured decision criterion (in cases where it is observed at decision time) or the predicted decision criterion (in cases where the manifest criterion is unobserved at decision time) is a good representation of the latent decision criterion. In the absence of bias, we could, for instance, assume that differences in measured medical spending between candidates correspond to equal differences between candidates in their medical need. In the absence of bias, it makes sense to allocate goods based on the measured (or the predicted) decision criterion.

A final point of discussion is the question whether distances between candidates should be defined on the group-level or the individual-level. Friedler et al. (2021) and the majority of fairness metrics (Mitchell et al., 2021) chose the group-level. This includes fairness metrics that measure differences in prediction errors between (a set of pre-defined) groups, such as overall accuracy equality (Berk et al., 2021) or equalized odds (Hardt et al., 2016). Bias is present if the transition between spaces (operationalization, measurement, or prediction) changes the distance between members of pre-defined social groups. The groups are defined based on so-called protected attributes, including amongst others gender, sexual orientation, disability, and ethnicity (Barocas and Selbst, 2016). Under most anti-discrimination laws, these protected attributes should not affect allocation decisions. Put differently, changes in the relative distance between individual candidates are only considered as bias when these changes align with protected groups. Consider again the probation panel that wants to allocate probation (the good) based on the number of re-offenses (manifest decision criterion), measured as the number of re-arrests (measured decision criterion). We noted that measurement bias increases the distance between Black persons and White persons in the measurement space compared to the indicator space. The group-level perspective would indeed recognize this measurement bias as a relevant form of bias. Now imagine that the measurement operation introduces a similar bias between left-handed and right-handed persons. For whatever reason, left-handed persons are re-arrested at higher rates than right-handed persons even if they have the same level of re-offending. Accordingly, the measurement would increase the distance between left-handed persons and right-handed persons in the measurement space compared to the indicator space. The group-level perspective would not recognize this as a relevant instance of bias because handedness is not a protected attribute.

In our opinion, this is a problematic implication of the group-level perspective. In general, we agree that some attributes are especially important because we need to redress historical injustices and because unequal treatment based on these attributes exists across a large range of allocation decisions (Loi et al., 2021). We argue, however, that bias that systematically affects groups defined by seemingly innocent attributes like handedness becomes problematic in the context of data-driven decision-making. Once data-driven decision-making is implemented by an institution, it is applied to a large number of candidates and regulates the access to the goods that the institution allocates. The institution frequently has the monopoly on the good, such that candidates have no other choice than to subject themselves to the data-driven decision process if they want the good. Job-seekers who seek access to support programs can only turn to the public employment agency, convicted persons can only turn to the probation panel if they want probation. In this situation, we believe, it would also be wrong if the decision process were systematically biased against left-handed persons (or any other group defined by non-protected attributes). More succinctly: If a data-driven decision process is applied to fully regulate the allocation of a good, it has the potential to create new groups that are systematically disadvantaged in the access to that good (Fazelpour and Lipton, 2020). These groups may be defined by protected attributes but can also be defined by any other non-protected attribute.

The individual-level perspective helps to address this point. The individual candidate is seen as the collection of all her attributes (protected and non-protected). Any change in the relative distance between candidates that aligns with one (or more) attribute of the candidates is a relevant instance of bias—irrespective of whether the attribute is protected or not. This is the spirit in which we formulated error fairness (Section 4). In the language of this section, error fairness states that the prediction operation is unbiased (or fair) if and only if the prediction does not change the relative distance between candidates in the prediction space compared to the measurement space. We acknowledge that error fairness is an aspirational target. To proof that a prediction operation is error fair requires showing that changes in the relative distance between candidates that occur in the transition from measurement space to prediction space are independent of all candidate attributes. The major problem is that we never observe all attributes of the candidates. Therefore, it is impossible to show that none of the candidate attributes is related to changes in relative distance. Again, substantive background knowledge and critical thinking are the best weapons to fight bias. We should strive to test (within the limits of privacy rights) associations between distance changes and the attributes that background knowledge and critical thinking suggest as the most important. We should not, however, limit ourselves to a pre-defined set of protected attributes. Protected attributes might be relevant in the case under study but they might just as well be irrelevant.

## 6. Discussion

The advent of data-driven decision-making in more and more areas of life (e.g., automated job advertisements, employee management, college admission, credit scoring, or more general access to public services) raises the dual problem of fairness in predictions and justice in decisions. Fairness and justice are conflated in the existent literature on data-driven decision-making systems, with the consequence that there exists a multitude of mutually incompatible fairness definitions—each motivated by a distinct set of moral concerns. To advance the literature, we propose an alternative approach that builds on a clean distinction between fairness and justice. Fairness regulates the distribution of prediction errors, whereas justice regulates the allocation of goods. The approach has practical implications for the design of data-driven decision systems but should also be viewed in light of its limitations.

### 6.1. Implications for practice

The approach suggests the following four-step process to designing fair and just decision systems. (a) Make a well-justified choice of the distributive justice principle. The principle is well-justified if a convincing rational defense of the principle can be provided to all candidates who are eventually affected by it. The design of an allocation system, therefore, requires stake-holder involvement—a requirement shared by impact assessment frameworks developed for data-driven decision systems (Selbst, 2018; Mantelero, 2018; Metcalf et al., 2021). (b) Make a context-fitting translation of the chosen justice principle(s) into an allocation principle. The allocation principle consists of a set of decision criteria and a rule that specifies how the criteria are related to allocation decisions. The translation is context-fitting if the chosen criterion and rule transport the general intention of the justice principle into the specific allocation context. (c) If the decision criterion is unobserved at decision time, use a fair prediction algorithm to predict its value. (d) Investigate whether the decision procedure is affected by operationalization bias, measurement bias, or prediction bias.

The approach highlights that fairness in predictions is one among multiple concerns. The selection of the distributive justice principle, its translation into an allocation principle, and the instrument that measures the decision criterion require equally close scrutiny. Note that the approach is modular: It is possible to reject our fairness definition and still accept our justice definition (and vice versa). A researcher who is not convinced by error fairness can apply her favored alternative fairness definition. The resulting predictions are then translated into an allocation decision *via* a well-justified allocation principle.

### 6.2. Limitations

While we believe that *error fairness* formalizes an intuitive and useful definition of fairness, its translation into a fairness metric proved rather difficult. We proposed the *R*^2^ from a linear regression of prediction errors on candidate features as a possible metric. The *R*^2^-metric is a necessary but not sufficient condition for error fairness: A prediction algorithm that satisfies error fairness achieves good results on the *R*^2^-metric. But the algorithm can violate error fairness and still achieve good results if the violation is due to systematic relationships between prediction errors and unobserved features. Future research should identify metrics with a stronger connection to error fairness.

Error fairness is not sensitive to historical bias (Suresh and Guttag, 2020). Historical bias is present if the data on which the prediction algorithm is trained reflect past discrimination against certain groups of candidates. Discrimination creates differences in bases rates: Members of the disadvantaged groups have less favorable values on the decision criterion. The prediction algorithm learns the historical bias and assigns less favorable predictions to members of the disadvantaged groups. Error fairness is not violated in the presence of historical bias as long as the predictions accurately reflect the true values of the decision criterion for all candidates. The predictions should track differences in base rates. Other fairness definitions (independence, counterfactual fairness) are sensitive to historical bias. We adopt the position of Corbett-Davies and Goel (2018) on this point: The fact that differences in base rates are a product of past discrimination does not mean that current predictions are inaccurate or that better societal outcomes could be achieved by altering predictions. More succinctly: “It would be misleading to characterize an algorithm or its training data as unfair for accurately identifying existing statistical patterns” (Corbett-Davies and Goel, 2018, p. 13). Importantly, we do not reject the need to correct unwanted discrimination or historical bias. Corrections should be applied at the decision step and not the prediction step, however. If there exists a justice principle that justifies such corrections in a given allocation problem (and we believe that there often is such a principle), it is permissible to define an allocation principle that implements the necessary corrections.

Finally, the list of *middle-range distributive justice principles* is not exhaustive. We invite researchers and practitioners to add to the list. To be admissible to the list, justice principles must define and justify (a) a decision criterion and (b) a rule that relates the criterion to the allocation of goods. The justice principle should not regulate the allocation of prediction errors. Equality of opportunity (Arneson, 2015) is a promising candidate. Equality of opportunity restricts the set of permissible decision criteria to criteria that are not related to protected features. Or else, it recommends allocation rules that compensate members of historically disadvantaged groups for discrimination that prevented them from developing the decision criterion.

## 7. Conclusion

Prior work on data-driven decision-making systems extensively explored the moral foundations of prominent algorithmic fairness definitions. This paper contributes a cleaner distinction between fairness and justice in data-driven decision-making. This distinction is instrumental for ethical self-assessment when building data-driven decision systems and can also guide regulations such as the EU AI Act. We clarify the relation between fairness and justice and provide clear definitions of both concepts. The paper provides an overview of distributive justice theories and a recipe for implementing the theories into the decision-making pipeline. Taken together, we contribute the outline of a principled local justice approach to the design of fair and just data-driven decision procedures—an approach that is urgently needed as data-driven decision-making increasingly enters all walks of life.

## Data availability statement

The original contributions presented in the study are included in the article/supplementary material, further inquiries can be directed to the corresponding author/s.

## Author contributions

All authors contributed equally to the conception and design of the study. MK and CK conducted the main conceptual analysis. MK wrote the first draft of the manuscript. CK wrote sections of the manuscript. All authors contributed significantly to manuscript revision and read and approved the submitted version.

## Funding

The work of MK was supported by the University of Mannheim's Graduate School of Economic and Social Sciences. We acknowledge funding from the Baden-Württemberg Stiftung (grant FairADM—Fairness in Algorithmic Decision Making) and the Volkswagen Stiftung (grant Consequences of Artificial Intelligence for Urban Societies). Part of this work was done while CK was visiting the Simons Institute for the Theory of Computing, UC Berkeley. The publication of this article was funded by the University of Mannheim.

## Conflict of interest

The authors declare that the research was conducted in the absence of any commercial or financial relationships that could be construed as a potential conflict of interest.

## Publisher's note

All claims expressed in this article are solely those of the authors and do not necessarily represent those of their affiliated organizations, or those of the publisher, the editors and the reviewers. Any product that may be evaluated in this article, or claim that may be made by its manufacturer, is not guaranteed or endorsed by the publisher.

## Footnotes

1 We make the simplifying assumption that the good is valued uniformly across all candidates. All candidates define the good either as a benefit or as a burden and candidates do not differ in the degree to which they value the good as either positive or negative. We believe that these simplifications are justified because real-world institutions typically lack information about inter-individual differences in the valuation of goods. Even if information on candidate preferences is available, its relevance as a decision criterion for the allocation is usually low.

2 We could also use an admission procedure (Elster, 1992) whereby each candidates whose value on the decision criterion surpasses a threshold value receives a unit of the good. Such a procedure, however, makes it difficult for an institution to plan because the number of candidates who surpass the threshold can fluctuate strongly over time.

3 In the categorical case, we are focusing on binary (or binarized) decision criteria.

4 Allocation principles produce (unintended) incentive effects when the principle is public knowledge and the decision-relevant criteria can be modified by the candidates. Positive incentive effects arise when attempts to acquire the decision-relevant criteria induce socially valuable behavior from the candidates (e.g., if kidney transplants are assigned preferably to non-smokers). Negative incentive effects occur when the induced behavior is socially harmful (e.g., if the military policy to draft only young men who can fire a gun induces young men to cut off their index fingers). Public knowledge of the decision-relevant criteria invites candidates to game the system by misrepresenting their private attributes.

5 Example: The outcome is the probability of re-employment among unemployed job-seekers. The good is participation in an active labor market training program that positively affects re-employment probability. The inequality metric is the Gini-coefficient applied to the distribution of post-allocation re-employment probability. The goal is to find the allocation of program participation to job-seekers that minimizes the Gini-coefficient. The allocation principle states that all candidates who receive program participation in this allocation scheme should actually receive program participation.

6 The leveling-down objection is best understood *via* an example. Let (A, B, C) be the outcomes for candidates A, B, and C that are generated by an allocation scheme. The choice is between two such allocation schemes: (1, 1, 1) and (10, 20, 20). The equal outcome principle, in its strictest sense, would force us to choose the first allocation scheme because it is clearly more equal than the second. The leveling-down objection is that we could make everyone better-off by choosing the second allocation scheme, even the candidate who receives the lowest outcome. The equal outcome principle forces us to make everyone worse-off than they could be even if this reduces inequality only by a small amount.

7 The estimates are based on a Gradient Boosting Machine trained on a sample of Computer Science researchers active from 1993 to 2016. For more information on the data, see Weihs and Etzioni (2017). More information on the prediction model and an evaluation of the prediction fairness can be found in Kuppler (2022).

8 Maximization applies if attainment of the outcome is beneficial. Minimization applies if the outcome is harmful. Example: With a given supply of kidney transplants, we wish to *maximize* the years of life saved. With a fixed supply of food, we wish to *minimize* hunger.

9 Continued example: We allocate the kidney transplants to the candidates in whom the transplant will produce the largest increment in life expectancy. We allocate the food to the candidates in whom the food will produce the largest increment in hunger reduction.

10 It is a generalization of the *Individual increments of welfare* criterion (Elster, 1992, p. 85) to outcomes that are not necessarily related to candidates' individual welfare.

11 We are indebted to an anonymous reviewer for suggesting mutual information.

12 Hertweck et al. (2021) present a similar four-space framework to identify the conditions under which egalitarian arguments support the application of statistical parity, a fairness metric that requires that predictions are statistically independent from protected attributes like gender and ethnicity. The framework presented in this paper has a different focus. It is not limited to egalitarian arguments and it is not geared at justifying a specific fairness metric.

## References

[B1] Adler M. D. Holtug N. (2019). Prioritarianism: a response to critics. Polit. Philos. Econ. 18, 101–144. 10.1177/1470594X19828022

[B2] Alikhademi K. Drobina E. Prioleau D. Richardson B. Purves D. Gilbert J. E. (2021). A review of predictive policing from the perspective of fairness. Artif. Intell. Law 30, 1–17. 10.1007/s10506-021-09286-4

[B3] Angwin J. Mattu S. Kirchner L. (2016). Machine Bias. Technical report, ProPublica.

[B4] Arneson R. (2013). “Egalitarianism,” in The Stanford Encyclopedia of Philosophy, ed E. N. Zalta (Stanford: Metaphysics Research Lab; Stanford University).

[B5] Arneson R. (2015). “Equality of opportunity,” in The Stanford Encyclopedia of Philosophy, ed E. N. Zalta (Stanford: Metaphysics Research Lab; Stanford University).

[B6] Barda N. Riesel D. Akriv A. Levy J. Finkel U. Yona G.. (2020). Developing a COVID-19 mortality risk prediction model when individual-level data are not available. Nat. Commun. 11:4439. 10.1038/s41467-020-18297-9 32895375 PMC7477233

[B7] Barocas S. Selbst A. D. (2016). Big data's disparate impact. Calif. Law Rev. 104, 671–732. 10.2139/ssrn.2477899

[B8] Berk R. Heidari H. Jabbari S. Kearns M. Roth A. (2021). Fairness in criminal justice risk assessments: the state of the art. Sociol. Methods Res. 50, 3–44. 10.1177/0049124118782533

[B9] Brock G. (2018). Sufficiency and Needs-Based Approaches, Vol. 1. New York, NY: Oxford University Press. 10.1093/oxfordhb/9780199645121.013.6

[B10] Carton S. Helsby J. Joseph K. Mahmud A. Park Y. Walsh J.. (2016). “Identifying police officers at risk of adverse events,” in 22nd ACM SIGKDD International Conference on Knowledge Discovery and Data Mining (Association for Computing Machinery), 67–76. 10.1145/2939672.2939698

[B11] Castro C. O'Brien D. Schwan B. (2021). “Fairness and machine fairness,” in Proceedings of the 2021 AAAI/ACM Conference on AI, Ethics, and Society, AIES '21 (New York, NY: Association for Computing Machinery), 446. 10.1145/3461702.3462577

[B12] Chouldechova A. (2016). Fair prediction with disparate impact: a study of bias in recidivism prediction instruments. arXiv:1610.07524 [cs, stat]. 10.1089/big.2016.0047 28632438

[B13] Cohen R. L. (1987). Distributive justice: theory and research. Soc. Just. Res. 1, 19–40. 10.1007/BF01049382

[B14] Corbett-Davies S. Goel S. (2018). The measure and mismeasure of fairness: a critical review of fair machine learning. arXiv: 1808.00023. 10.48550/arXiv.1808.00023

[B15] Cover T. M. Thomas J. A. (2006). Elements of Information Theory, 2nd Edn. Hoboken, NJ: Wiley-Interscience.

[B16] Desiere S. Langenbucher K. Struyven L. (2019). “Statistical profiling in public employment services: an international comparison,” in OECD Social, Employment and Migration Working Papers (Paris), 224.

[B17] Deutsch M. (1975). Equity, equality, and need: what determines which value will be used as the basis of distributive justice? J. Soc. Issues 31, 137–149. 10.1111/j.1540-4560.1975.tb01000.x

[B18] Dworkin R. (1981). What is equality? Part 1: equality of welfare. Philos. Publ. Affairs 10, 185–246.

[B19] Elster J. (1992). Local Justice: How Institutions Allocate Scarce Goods and Necessary Burdens. New York, NY: Russell Sage Foundation.

[B20] Engler A. (2022a). The EU AI Act Will Have Global Impact, But a Limited Brussels Effect. Technical report. Brookings Institute.

[B21] Engler A. (2022b). Institutionalizing Data Analysis in German Federal Governance. Technical report. Brookings Institute.

[B22] Fazelpour S. Lipton Z. C. (2020). “Algorithmic fairness from a non-ideal perspective,” in Proceedings of the AAAI/ACM Conference on AI, Ethics, and Society (New York, NY: ACM), 57–63. 10.1145/3375627.3375828

[B23] Feldman F. Skow B. (2020). “Desert,” in The Stanford Encyclopedia of Philosophy, ed E. Zalta (Stanford: Metaphysics Research Lab; Stanford University).

[B24] Friedler S. A. Scheidegger C. Venkatasubramanian S. (2021). The (im)possibility of fairness: different value systems require different mechanisms for fair decision making. Commun. ACM 64, 136–143. 10.1145/3433949

[B25] Gosepath S. (2011). “Equality,” in The Stanford Encyclopedia of Philosophy, ed E. N. Zalta (Stanford: Metaphysics Research Lab; Stanford University).

[B26] Hardt M. Price E. Srebro N. (2016). Equality of opportunity in supervised learning. arXiv:1610.02413 [cs]. 10.48550/arXiv.1610.02413

[B27] Hart N. Yohannes M. (2019). Evidence Works: Cases Where Evidence Meaningfully Informed Policy. Bipartisan Policy Center.

[B28] Hebert-Johnson U. Kim M. Reingold O. Rothblum G. (2018). “Multicalibration: calibration for the (computationally-identifiable) masses,” in Proceedings of the 35th International Conference on Machine Learning, Vol. 80 of Proceedings of Machine Learning Research, eds J. Dy and A. Krause (Stockholm), 1939–1948.

[B29] Heidari H. Loi M. Gummadi K. P. Krause A. (2019). “A moral framework for understanding fair ML through economic models of equality of opportunity,” in Proceedings of the Conference on Fairness, Accountability, and Transparency (Atlanta, GA), 181–190. 10.1145/3287560.3287584

[B30] Hertweck C. Heitz C. Loi M. (2021). “On the moral justification of statistical parity,” in Proceedings of the 2021 ACM Conference on Fairness, Accountability, and Transparency (ACM), 747–757. 10.1145/3442188.3445936

[B31] Hirsch J. E. (2005). An index to quantify an individual's scientific research output. Proc. Natl. Acad. Sci. U.S.A. 102, 16569–16572. 10.1073/pnas.0507655102 16275915 PMC1283832

[B32] Holtug N. (2007). “Prioritarianism,” in Egalitarianism: New Essays on the Nature and Value of Equality, eds N. Holtug and K. Lippert-Rasmussen (Oxford; New York, NY: Clarendon Press), 125–156.

[B33] Kim M. P. Ghorbani A. Zou J. (2019). “Multiaccuracy: black-box post-processing for fairness in classification,” in Proceedings of the 2019 AAAI/ACM Conference on AI, Ethics, and Society, AIES '19 (New York, NY: Association for Computing Machinery), 247–254. 10.1145/3306618.3314287

[B34] Kleinberg J. Mullainathan S. Raghavan M. (2016). Inherent trade-offs in the fair determination of risk scores. arXiv: 1609.05807. 10.48550/arXiv.1609.05807

[B35] Konow J. (2003). which is the fairest one of all? A positive analysis of justice theories. J. Econ. Liter. 41, 1188–1239. 10.1257/002205103771800013

[B36] Kozodoi N. Jacob J. Lessmann S. (2021). Fairness in credit scoring: assessment, implementation and profit implications. Eur. J. Oper. Res. 297, 1083–1094. 10.1016/j.ejor.2021.06.023

[B37] Kuppler M. (2022). Predicting the future impact of Computer Science researchers: is there a gender bias? Scientometrics. 10.1007/s11192-022-04337-2. [Epub ahead of print].

[B38] Lamont J. Favor C. (2017). “Distributive justice,” in The Stanford Encyclopedia of Philosophy, ed E. N. Zalta (Stanford: Metaphysics Research Lab; Stanford University). 10.4324/9781315257563

[B39] Lepri B. Oliver N. Letouzé E. Pentland A. Vinck P. (2018). Fair, transparent, and accountable algorithmic decision-making processes: the premise, the proposed solutions, and the open challenges. Philos. Technol. 31, 611–627. 10.1007/s13347-017-0279-x

[B40] Lippert-Rasmussen K. (2018). “Justice and bad luck,” in The Stanford Encyclopedia of Philosophy, ed E. N. Zalta (Stanford: Metaphysics Research Lab; Stanford University).

[B41] Loi M. Herlitz A. Heidari H. (2021). “Fair equality of chances for prediction-based decisions,” in Proceedings of the 2021 AAAI/ACM Conference on AI, Ethics, and Society, AIES '21 (New York, NY: Association for Computing Machinery), 756. 10.1145/3461702.3462613

[B42] Lum K. Isaac W. (2016). To predict and serve? Significance 13, 14–19. 10.1111/j.1740-9713.2016.00960.x

[B43] Makhlouf K. Zhioua S. Palamidessi C. (2020). On the applicability of ML fairness notions. arXiv:2006.16745 [cs, stat]. 10.48550/arXiv.2006.16745

[B44] Mantelero A. (2018). AI and big data: a blueprint for a human rights, social and ethical impact assessment. Comput. Law Secur. Rev. 34, 754–772. 10.1016/j.clsr.2018.05.017

[B45] Mehrabi N. Morstatter F. Saxena N. Lerman K. Galstyan A. (2019). A survey on bias and fairness in machine learning. arXiv:1908.09635 [cs]. 10.48550/arXiv.1908.09635

[B46] Metcalf J. Moss E. Watkins E. A. Singh R. Elish M. C. (2021). “Algorithmic impact assessments and accountability: the co-construction of impacts,” in Proceedings of the 2021 ACM Conference on Fairness, Accountability, and Transparency (ACM), 735–746. 10.1145/3442188.3445935

[B47] Metz C. Satariano A. (2020). An Algorithm That Grants Freedom, or Takes It Away. New York Times.

[B48] Mitchell S. Potash E. Barocas S. D'Amour A. Lum K. (2021). Algorithmic fairness: choices, assumptions, and definitions. Annu. Rev. Stat. Appl. 8, 141–163. 10.1146/annurev-statistics-042720-125902

[B49] Moriarty J. (2018). Desert-Based Justice, Vol. 1. New York, NY: Oxford University Press. 10.1093/oxfordhb/9780199645121.013.7

[B50] New Zealand Ministry of Social Development (2014). The Feasibility of Using Predictive Risk Modelling to Identify New-Born Children Who Are High Priority for Prevention Services. Ministry of Social Development, Wellington.

[B51] Nozick R. (1974). Anarchy, State, and Utopia. Basic Books, a Member of the Perseus Books Group, New York, NY.

[B52] Obermeyer Z. Powers B. Vogeli C. Mullainathan S. (2019). Dissecting racial bias in an algorithm used to manage the health of populations. Science 366, 447–453. 10.1126/science.aax2342 31649194

[B53] Parfit D. (1997). Equality and priority. Ratio 10, 202–221. 10.1111/1467-9329.00041

[B54] Rawls J. (1971). A Theory of Justice. Cambridge: Harvard University Press. 10.4159/9780674042605

[B55] Rodolfa K. T. Saleiro P. Ghani R. (2021). “Bias and fairness,” in Big Data and Social Science: Data Science Methods and Tools for Research and Practice, 2nd Edn, eds I. Foster, R. Ghani, R. S. Jarmin, F. Kreuter, and J. Lane (Boca Raton, FL: CRC Press), 281–312.

[B56] Schmidt V. H. (1992a). Adaptive justice: local distributive justice in sociological perspective. Theory Soc. 21, 789–816. 10.1007/BF00992812

[B57] Schmidt V. H. (1992b). Lokale gerechtigkeit: perspektiven soziologischer gerechtigkeitsanalyse. Zeitsch. Soziol. 21, 3–15. 10.1515/zfsoz-1992-0101

[B58] Schmidt V. H. (1994). Bounded justice. Soc. Sci. Inform. 33, 305–333. 10.1177/053901894033002009

[B59] Selbst A. D. (2018). Disparate impact in big data policing. Georgia Law Rev. 52. 10.2139/ssrn.2819182

[B60] Sinnott-Armstrong W. (2021). “Consequentialism,” in The Stanford Encyclopedia of Philosophy, ed E. N. Zalta (Stanford: Metaphysics Research Lab; Stanford University).

[B61] Suresh H. Guttag J. V. (2020). A framework for understanding unintended consequences of machine learning. arXiv:1901.10002. 10.48550/arXiv.1901.10002

[B62] Törnblom K. Kazemi A. (2015). “Distributive justice,” in The Oxford Handbook of Justice in the Workplace, eds R. S. Cropanzano and M. L. Ambrose (New York, NY: Oxford University Press), 15–50.

[B63] Weihs L. Etzioni O. (2017). “Learning to predict citation-based impact measures,” in Proceedings of the 17th ACM/IEEE Joint Conference on Digital Libraries, JCDL '17 (IEEE Press), 49–58. 10.1109/JCDL.2017.7991559

[B64] Završnik A. (2021). Algorithmic justice: algorithms and big data in criminal justice settings. Eur. J. Criminol. 18, 623–642. 10.1177/1477370819876762

